# Preserving Honey

**Published:** 1871-08

**Authors:** 


### PRESERVING HONEY.

Let it run through a fine sieve to get out all the wax, boil it gently in an earthen vessel, skim off the lightish foam which gathers on the top, cool in jars, tightly cover them, and put them in a cool place.

As honey is a luxury on every table, and a healthful article of food the world over, a process for keeping it the year round, in all its purity, should be known and practiced in every family so well to do as to make their own honey.

Next to the wonder that every farmer does not have a fruit orchard, is that honey should not be made a product in every country household.

